# Association of medial meniscal extrusion with medial tibial osteophyte distance detected by T2 mapping MRI in patients with early-stage knee osteoarthritis

**DOI:** 10.1186/s13075-017-1411-0

**Published:** 2017-09-12

**Authors:** Shinnosuke Hada, Muneaki Ishijima, Haruka Kaneko, Mayuko Kinoshita, Lizu Liu, Ryo Sadatsuki, Ippei Futami, Anwajan Yusup, Tomohiro Takamura, Hitoshi Arita, Jun Shiozawa, Takako Aoki, Yuji Takazawa, Hiroshi Ikeda, Shigeki Aoki, Hisashi Kurosawa, Yasunori Okada, Kazuo Kaneko

**Affiliations:** 1 Department of Medicine for Orthopaedics and Motor Organ, Juntendo University Graduate School of Medicine, 2-1-1, Hongo, Bunkyo-ku, Tokyo, 113-8421 Japan; 2 Department of Pathophysiology for Locomotive and Neoplastic Diseases, Juntendo University Graduate School of Medicine, 2-1-1 Hongo, Bunkyo-ku, Tokyo, 113-8421 Japan; 3 Sportology Center, Juntendo University Graduate School of Medicine, Tokyo, Japan; 4 Research Institute for Diseases of Old Age, Juntendo University Graduate School of Medicine, Tokyo, Japan; 5 Department of Radiology, Juntendo University Graduate School of Medicine, Tokyo, Japan; 6 Department of Orthopaedics, Juntendo Tokyo Koto Geriatric Medical Center, Tokyo, Japan

**Keywords:** Osteoarthritis, Early-stage knee osteoarthritis, Medial meniscal extrusion, Diagnosis, MRI, Osteophyte, T2 mapping

## Abstract

**Background:**

Medial meniscal extrusion (MME) is associated with progression of medial knee osteoarthritis (OA), but no or little information is available for relationships between MME and osteophytes, which are found in cartilage and bone parts. Because of the limitation in detectability of the cartilage part of osteophytes by radiography or conventional magnetic resonance imaging (MRI), the rate of development and size of osteophytes appear to have been underestimated. Because T2 mapping MRI may enable us to evaluate the cartilage part of osteophytes, we aimed to examine the association between MME and OA-related changes, including osteophytes, by using conventional and T2 mapping MRI.

**Methods:**

Patients with early-stage knee OA (*n* = 50) were examined. MRI-detected OA-related changes, in addition to MME, were evaluated according to the Whole-Organ Magnetic Resonance Imaging Score. T2 values of the medial meniscus and osteophytes were measured on T2 mapping images. Osteophytes surgically removed from patients with end-stage knee OA were histologically analyzed and compared with findings derived by radiography and MRI.

**Results:**

Medial side osteophytes were detected by T2 mapping MRI in 98% of patients with early-stage knee OA, although the detection rate was 48% by conventional MRI and 40% by radiography. Among the OA-related changes, medial tibial osteophyte distance was most closely associated with MME, as determined by multiple logistic regression analysis, in the patients with early-stage knee OA (β = 0.711, *p* < 0.001). T2 values of the medial meniscus were directly correlated with MME in patients with early-stage knee OA, who showed ≥ 3 mm of MME (*r* = 0.58, *p* = 0.003). The accuracy of osteophyte evaluation by T2 mapping MRI was confirmed by histological analysis of the osteophytes removed from patients with end-stage knee OA.

**Conclusions:**

Our study demonstrates that medial tibial osteophyte evaluated by T2 mapping MRI is frequently observed in the patients with early-stage knee OA, showing close association with MME, and that MME is positively correlated with the meniscal degeneration.

## Background

Osteoarthritis of the knee (knee OA) is an important public health concern because the prevalence of the disease is increasing with the aging population, and to date there are no disease-modifying treatments [1]. An essential component of the knee joint is the meniscus, which functions as a shock absorber of local mechanical stress and maintains joint stability [2]. Dislocation and/or tears of the meniscus contribute to the development of knee OA [3]. In fact, previous studies have shown that medial meniscal extrusion (MME) [4], which is also called *medial meniscus subluxation* or *displacement* [5], is associated with the cartilage damage and disease progression of knee OA [6].

Osteophytes, one of the structural signs of knee OA, develop at the peripheries of the articular cartilage by following the processes of the endochondral ossification [7]. Osteophyte formation is generally considered to be a repair process of damaged articular cartilage and thus a late event in knee OA [7, 8]. Histologically, osteophytes are composed of the cartilage and bone parts [7, 9], but only the bone part has been clinically evaluated because the cartilage component is undetectable by radiography and difficult to evaluate by proton density-weighted magnetic resonance imaging (conventional MRI). These diagnostic methods, therefore, appear to have underestimated the rate of development and the size of osteophytes. On one hand, because T2 mapping MRI can monitor changes in water content and organization of the collagen network in articular cartilage [10], this diagnostic method is expected to be useful for detection and monitoring of the cartilage part of osteophytes in knee OA joints. On the other hand, a recent community cohort study of people without radiographic knee OA showed that osteophytes were detected by conventional MRI in 70–80% of middle-aged and old people (68%, 78%, and 80% of the people in their 50s, 60s, and 70s, respectively) [11]. These finding suggests that osteophyte formation is a common event in knee joints of aged people and may be involved in early-stage OA changes. However, the prevalence of osteophytes in patients with early-stage knee OA and relationships between osteophyte formation and MME remain elusive.

In the present study, we hypothesized that a positive relationship must be present between osteophyte formation and MME in early-stage knee OA. Thus, the purpose of this study was to examine the relationship of osteophytes and MME by introducing T2 mapping MRI to analyze OA changes in patients with early-stage knee OA. We evaluated osteophytes by measuring both cartilage and bone parts using T2 mapping MRI in patients with early-stage knee OA, and we analyzed the relationship between MME and osteophytes. We also assessed the accuracy of the T2 mapping MRI data in a combined histological study of the osteophytes. Our data demonstrate that formation of medial tibial osteophytes is a change closely associated with MME in early-stage knee OA.

## Methods

### Subjects

The sample size of this study was determined by the number of patients who had the following conditions during the study period (May 2012 to September 2015). Five hundred forty-five patients with knee pain visited the outpatient clinic of the authors (SH, MI, and HaK) in Juntendo University Hospital to seek therapy and underwent radiography and conventional and T2 mapping MRI during the study period. Diagnosis of knee OA was made according to the American College of Rheumatology criteria [12] and MRI criteria [13]. When the patients showed grade 0, 1, or 2 of the Kellgren-Lawrence (K/L) classification [14, 15] and fulfilled the definition of OA based on MRI criteria [13], they were classified as having early-stage knee OA. Fifty of the 545 patients had early-stage knee OA, and they were enrolled in this study. Among patients with end-stage knee OA (*n* = 4, three females and one male, average age 72.7 years, body mass index 23.2 kg/cm^2^) who underwent total knee arthroplasty, the findings of histology, radiographs, and MRI scans of medial tibial osteophytes were compared.

The study protocol, which complied with the principles outlined in the Declaration of Helsinki, was approved by the Ethical Committee Review Board at Juntendo University. Written informed consent was obtained from the patients with end-stage knee OA. Because the studies of patients with early-stage knee OA were categorized as retrospective, the Ethical Committee Review Board waived the requirement for patients’ informed consent because of the anonymous nature of the data.

### Radiographic evaluation

Standing, extended, and anteroposterior and lateral view radiographs, as well as posteroanterior weight-bearing radiographs made with the knee in 45 degrees of flexion, were evaluated according to K/L grade [14, 15].

### MRI analyses

The knee joints were analyzed using the MAGNETOM Verio MR 3.0-Tesla MRI system (Siemens Medical Solutions, Erlangen, Germany) with proton density-weighted image fast spin echo sequences (repetition time [TR] 1800 milliseconds, echo time [TE] 11 milliseconds, slice thickness 3 mm, field of view [FOV] 160 mm, matrix 384 × 384) and T2 mapping sequences (TR 1000 milliseconds; TE 13.8, 27.6, 41.4, 55.2, and 69.0 milliseconds; slice thickness 3 mm; FOV 160 mm; matrix 384 × 384) as we previously described [16, 17]. The MRI data were evaluated in the medial tibiofemoral joint, and OA morphological changes were scored according to the Whole-Organ Magnetic Resonance Imaging Score (WORMS) [18]. Each region of a compartment received its own score, and these scores were added together [16]. The diagnosis of knee OA for the subjects was made using the MRI findings according to a previously reported method [16, 19]. The regions of interest on T2 mapping MRI studies were manually drawn using a three-dimensional image analysis software program (VirtualPlace; AZE, Tokyo, Japan). T2 values of the medial meniscus were calculated as described previously [16].

### Evaluation of MME and osteophytes

**Fig. 1:**
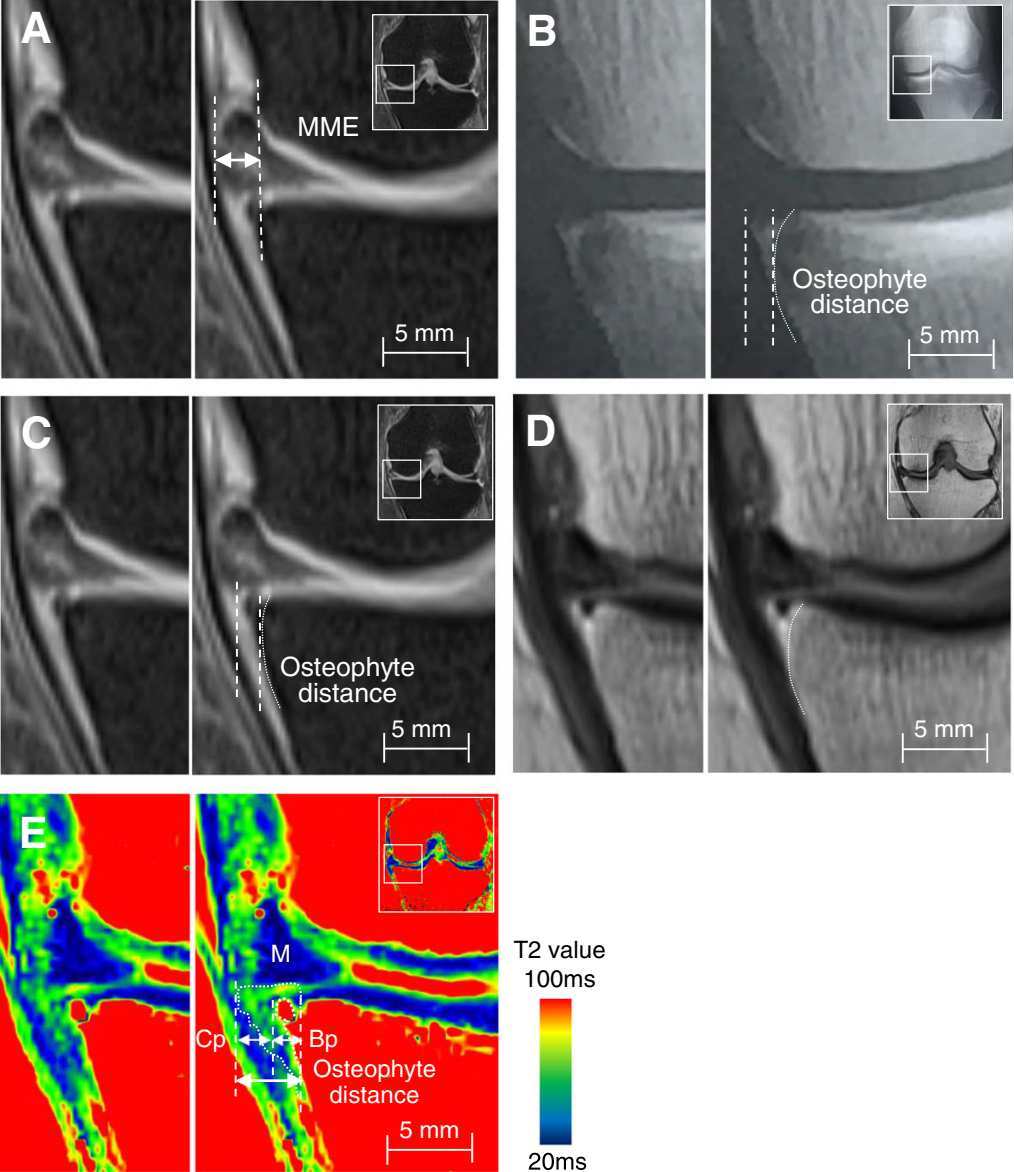
Representative data derived from the measurement of medial meniscal extrusion (MME) and medial tibial osteophyte distance by radiography, proton density-weighted magnetic resonance imaging (MRI), and T2 mapping MRI in the early-stage knee osteoarthritis joint. **a** Coronal fast spin echo proton density-weighted MRI with fat suppression. MME distance was measured as the distance from the outermost edge of the medial meniscus to a line connecting the femoral and tibial cortices. **b** Osteophyte distance measurement using radiography. **c** Osteophyte distance measurement using proton density-weighted MRI with fat suppression. **d** Definition of a boundary between the tibial cortex and osteophyte (high signal zone) using proton density-weighted imaging without fat suppression. **e** Osteophyte distance measurement using T2 mapping MRI. *Dashed lines* indicate cartilage and bone parts of the osteophyte. The line between bone part of osteophyte and tibial cortex was drawn by tracing the image obtained by proton density-weighted MRI without fat suppression. *Bp* Bone part of osteophyte, *Cp* Cartilage part of osteophyte, *M* Meniscus

MME distance was defined as the distance from the outermost edge of the medial meniscus to a line connecting the femoral and tibial cortices [3] and graded according to previously described methods [19]. Thus, the knee joint was examined by proton density-weighted MRI (conventional MRI) and MME distance was measured by drawing a line connecting the femoral and tibial cortices, as shown in Fig. 1a. Although an osteophyte is composed of bone and cartilage parts [7], only the bone part of the osteophyte was detected by radiography (Fig. 1b) and conventional MRI with or without suppression of fat signals (Fig. 1c and d). Therefore, we evaluated osteophytes by T2 mapping MRI, which enabled us to detect both cartilage and bone parts of osteophyte (Fig. 1e). Because the boundary between bone parts of the osteophyte and tibial cortex was difficult to determine by T2 mapping MRI, the borderline between the osteophyte and the tibial cortex was drawn by tracing identical slices of the knee on the conventional MRI scan and the T2 mapping MRI scan. The distance of the cartilage and bone parts of the osteophyte was calculated separately on T2 mapping images (Fig. 1e). The occurrence rate of osteophytes in the medial side of the knee joint was evaluated by radiography, conventional MRI, and T2 mapping MRI, in which positive osteophyte was defined as radiographic K/L grade ≤ 2 and WORMS grade ≤ 2 as previously reported [11].

### Histological examination

The medial tibial osteophytes obtained at surgery from patients with end-stage knee OA were fixed in 10% neutral buffered formalin. After decalcification, the osteophytes were cut vertically in the center from the outer border to the bottom. Serial paraffin sections were stained with hematoxylin and eosin and Safranin O and microscopically photodocumented. Distances of the osteophytes were determined by measuring the ossified and cartilage-capped areas using cellSens software (Olympus, Tokyo, Japan).

### Reproducibility measurements

Two observers (SH and TT) conducted the examination to assess interobserver reproducibility. The intraobserver reproducibility of T2 value measurement, WORMS evaluation, MME distance, and osteophyte distance by MRI was as follows: interclass correlation coefficient (ICC) for interreader agreement 0.91 (95% CI 0.64–0.98) for T2 value, 0.94 (95% CI 0.91–0.96) for WORMS, 0.95 (95% CI 0.78–0.99) for MME, and 0.94 (95% CI 0.85–0.98) for osteophyte distance. Observers were blinded to any patient information during the evaluation process. The interobserver reproducibility was also high: ICC 0.91 (95% CI 0.63–0.97) for T2 value measurement, 0.90 (95% CI 0.86–0.93) for WORMS, 0.93 (95% CI 0.76–0.98) for MME, and 0.94 (95% CI 0.84–0.97) for osteophyte distance. The weighted kappa coefficients of inter- and intraobserver reliability for WORMS were 0.66 and 0.65, respectively.

### Statistical analysis

All analyses were performed using IBM SPSS Statistics 21.0 software program (IBM, Armonk, NY, USA). Associations between MME- and OA-related factors, including cartilage, osteophyte, bone marrow lesion, subchondral bone cyst, subchondral bone attrition, medial meniscus lesion, medial femoral osteophyte distance, medial tibial osteophyte distance, and T2 value of medial meniscus, were determined by logistic regression and multiple logistic regression analyses. Relationships between tibial osteophyte distance and MME grade were assessed by one-way analysis of variance. Correlations between MME and T2 value of the medial meniscus were examined using Spearman’s correlation coefficients. *p* values < 0.05 were considered significant.

## Results

### Frequent detection of osteophytes composed of bone and cartilage parts by T2 mapping MRI in patients with early-stage knee OA

OA changes of knee joints in patients with early-stage OA (*n* = 50) were examined by radiography, conventional MRI, and T2 mapping MRI according to the methods depicted in Fig. 1. Characteristics of the patients and the findings of the medial tibiofemoral joint are summarized in Table 1. Although more than half of patients with early-stage knee OA (30 of 50 patients) showed radiographic K/L grade 0 or 1, OA changes were observed by conventional MRI (Table 1). MME distance in the patients was 3.0 ± 1.6 mm by conventional MRI (Table 1). When MME distance (Fig. 1a) and osteophyte distance (Fig. 1c) were assessed by conventional MRI, the MME distance appeared to be longer than that of osteophyte distance. However, osteophyte distance measured by T2 mapping MRI (Fig. 1e) (i.e., sum of the bone and cartilage parts) appeared to be similar to MME distance calculated by conventional MRI (3.3 ± 1.9 mm versus 3.0 ± 1.6 mm) (Table 1). Conventional MRI showed tears of the medial meniscus in 30% of the patients (15 of 50 patients), but no root tear was observed (Table 1).

**Table 1:** Characteristics of patients with early-stage knee osteoarthritis

Characteristics	Data
Number of subjects	50
Age, years	57.0 (15.1)
BMI, kg/cm^2^	24.1 (3.9)
Sex, M/F	23/27
Radiography	
K/L grade 0/1/2	3/27/20
Femorotibial angle, degrees	178.4 (2.6)
Medial joint space width, mm	3.7 (0.9)
Proton density-weighted MRI (MTFJ)	
WORMS	
Cartilage (0–30)	11.9 (6.8)
Osteophyte (0–35)	4.4 (5.1)
Bone marrow lesion (0–15)	1.0 (1.9)
Subchondral bone cyst (0–15)	2.5 (4.4)
Subchondral bone attrition (0–15)	1.3 (1.5)
Medial meniscus lesion (0–6)	2.2 (1.6)
Medial meniscus	
MME, mm	3.0 (1.6)
Tears, *n*	15
Root tear, *n*	0
T2 mapping MRI (MTFJ)	
T2 value of medial meniscus	22.0 (2.8)
Medial femoral osteophyte distance	
Bone part + cartilage part, mm	2.1 (1.4)
Bone part, mm	1.3 (0.9)
Cartilage part, mm	0.8 (0.8)
Medial tibial osteophyte distance	
Bone part + cartilage part, mm	3.3 (1.9)
Bone part, mm	1.8 (1.4)
Cartilage part, mm	1.5 (1.5)
Osteophyte prevalence (MTFJ)	
Radiograph	40.0%
Proton density-weighted MRI	48.0%
K/L 0	0%
K/L 1	44.4%
K/L 2	60.0%
T2 mapping MRI	98.0%
K/L 0	100%
K/L 1	96.7%
K/L 2	100%

*Abbreviations: BMI* Body mass index, *K/L* Kellgren-Lawrence classification, *MFTJ* Medial tibiofemoral joint, *MME* Medial meniscal extrusion, *MRI* Magnetic resonance imaging, *WORMS* Whole-Organ Magnetic Resonance Imaging Score

The femorotibial angle and the medial joint space width of the knee joints were determined at the center point of the medial femorotibial compartment on a radiograph. Osteophytes observed by radiograph and MRI were defined as the presence of K/L grade ≤ 2 and the presence of WORMS grade ≤ 2, respectively. The data are expressed as mean (SD), count, or percent

On one hand, the prevalence of osteophyte in the medial side of the knee joint of the patients was 40% by radiography, and conventional MRI showed osteophytes in 48% of the patients (Table 1). On the other hand, T2 mapping MRI demonstrated osteophytes in 98% of patients with early-stage knee OA, and the prevalence of osteophytes was increased depending on the radiographic severity of knee OA (Table 1). When medial tibial and femoral osteophyte distances in the patients were calculated by separately measuring the cartilage and bone parts using T2 mapping MRI, the sums of both parts in the medial femoral and tibial osteophyte distances were 2.1 ± 1.4 mm and 3.3 ± 1.9 mm, respectively (Table 1).

### Association between MME and medial tibial osteophyte distance in patients with early-stage knee OA

Associations between MME and MRI-detected OA features of the medial tibiofemoral joint were next examined. By univariate analysis, cartilage scores (β = 0.472), osteophyte (β = 0.350), subchondral bone cyst (β = 0.393), subchondral bone attrition (β = 0.300), medial meniscus lesion (β = 0.445), medial femoral osteophyte distance (β = 0.429), medial tibial osteophyte distance (β = 0.711) and T2 value of medial meniscus (β = 0.397) were associated with MME (Table 2). Multivariate analysis indicated that among these factors, the medial tibial osteophyte distance was most closely associated with MME (β = 0.711, *p* < 0.001) (Table 2).

**Table 2:** Association between medial meniscal extrusion and magnetic resonance imaging-detected osteoarthritis features of medial tibiofemoral joint of the knee in patients with early-stage knee osteoarthritis

Factor	Univariable β	*p* Value	Multivariable β	*p* Value
Proton density-weighted MRI				
WORMS				
Cartilage	0.472	0.001	0.352	0.009
Osteophyte	0.350	0.013	−0.200	0.207
Bone marrow lesion	0.112	0.440	−0.037	0.725
Subchondral bone cyst	0.393	0.005	−0.038	0.785
Subchondral bone attrition	0.300	0.034	−0.039	0.763
Medial meniscus lesion	0.445	0.001	0.174	0.144
T2 mapping MRI				
Medial femoral osteophyte distance	0.429	0.002	−0.070	0.648
Medial tibial osteophyte distance	0.711	<0.001	0.711	<0.001
T2 value of medial meniscus	0.397	0.004	0.080	0.489

*MRI* Magnetic resonance imaging, *WORMS* Whole-Organ Magnetic Resonance Imaging Score

Osteophyte distance by T2 mapping is shown as the sum of the bone and cartilage parts of osteophyte distance

**Fig. 2:**
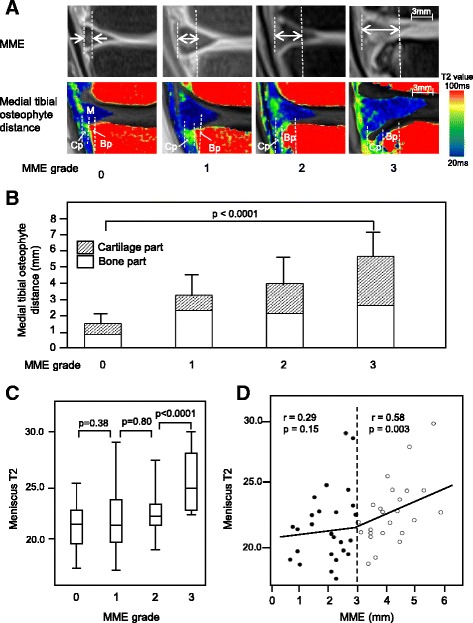
Association between medial meniscal extrusion (MME) and medial tibial osteophyte distance in patients with early-stage knee osteoarthritis. **a** Representative images of proton density-weighted magnetic resonance imaging (MRI) with fat suppression and T2 mapping MRI of the medial tibiofemoral joint. The patients were classified into four subgroups with MME grade 0, 1, 2, or 3. **b** Correlation between medial tibial osteophyte distance and MME. The medial tibial osteophyte distance was compared among the subgroups with MME grade 0 (*n* = 13), 1 (*n* = 13), 2 (*n* = 18), or 3 (*n* = 6). **c** Comparison of T2 values of medial meniscus among the subgroups with MME grade 0 (*n* = 13), 1 (*n* = 13), 2 (*n* = 18), or 3 (*n* = 6). **d** Correlation between T2 values of medial meniscus and MME. *Closed circles* represent the patient subgroup with MME grade 0 or 1 (*n* = 26); *open circles* represent the patients with MME grade 2 or 3 (*n* = 24). *Bp* Bone part of osteophyte, *Cp* Cartilage part of osteophyte, *M* Medial meniscus

Association between MME and medial tibial osteophyte distance was analyzed by dividing the subjects into four subgroups with MME grade 0 (distance < 2 mm), grade 1 (2–2.9 mm), grade 2 (3–4.9 mm), or grade 3 (≥5 mm) according to the degree of MME [20] (Fig. 2a). The medial tibial osteophyte distance (sum of the bone and cartilage parts of the osteophyte) in each subgroup positively correlated with the MME grades (*p* < 0.0001) (Fig. 2b). A similar positive correlation was also obtained when the distance of the cartilage part of the medial tibial osteophyte was separately evaluated (*p* = 0.0002). When the correlation between T2 values of the medial meniscus and MME was analyzed among the patient subgroups, the T2 value in the subgroup with MME grade 3 was significantly higher than that in the subgroup with grade 2 (*p* < 0.0001), whereas no significant differences were observed between the subgroups with MME grade 0, 1, or 2 (Fig. 2c). The patient subgroups with MME grade 2 or 3 showed a direct correlation with the T2 value of the medial meniscus (*r* = 0.58, *p* = 0.003), whereas no such association was obtained in the subgroups of MME grade 0 or 1 (*r* = 0.29, *p* = 0.15) (Fig. 2d).

### Histological demonstration of accuracy for medial tibial osteophyte distance evaluated by T2 mapping MRI

**Fig. 3:**
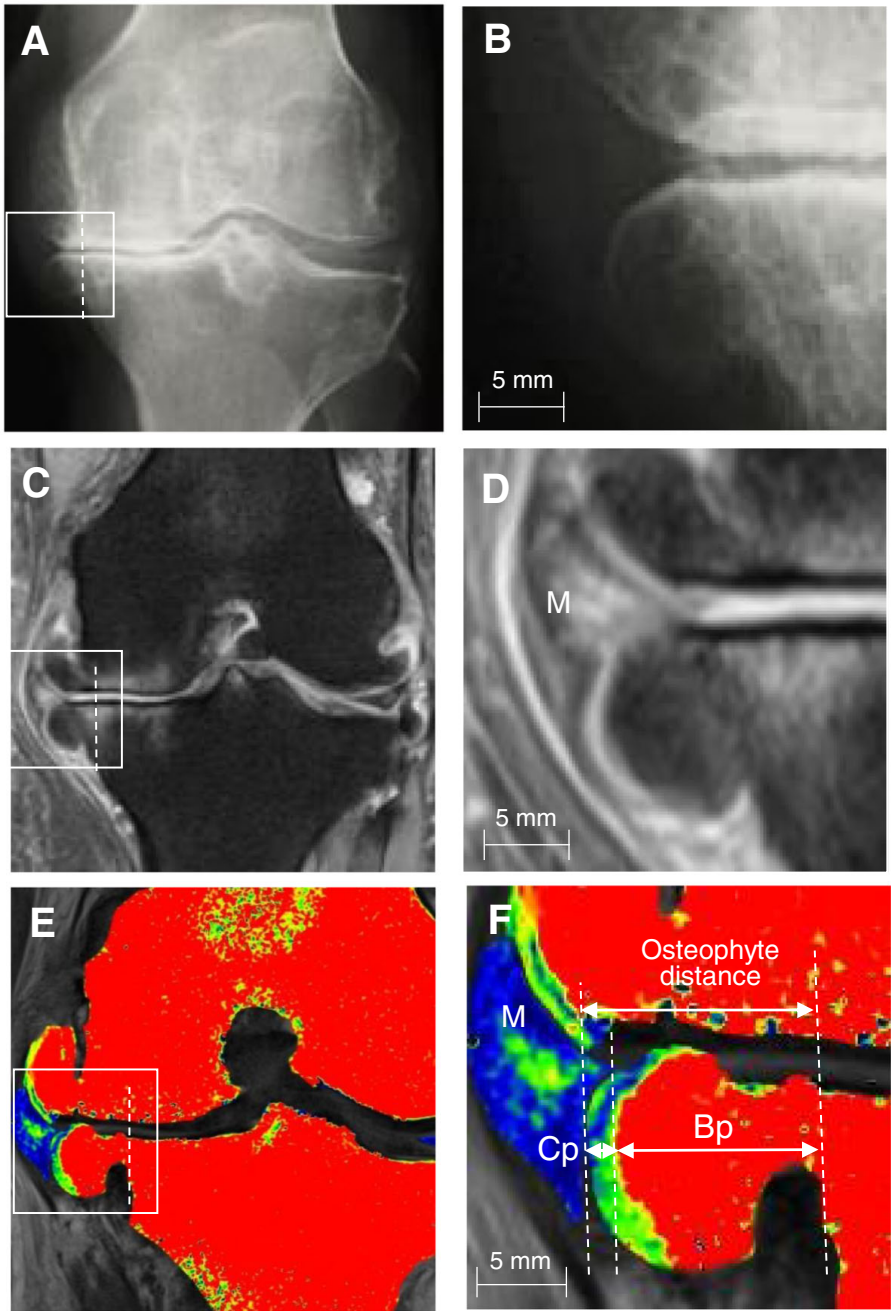
Findings of medial tibial osteophytes by radiography, proton density-weighted magnetic resonance imaging (MRI), and T2 mapping MRI. **a** Representative radiograph of the end-stage knee osteoarthritis joint. **b** High-power view of the boxed area in (**a**). **c** Proton density-weighted MRI with fat suppression of the same knee joint shown in (**a**). **d** High-power view of the boxed area in (**c**). **e** T2 mapping MRI of the same knee joint shown in (**a**). **f** High-power view of the boxed area in (**e**). *Bp* bone part of osteophyte, *Cp* cartilage part of osteophyte, *M* medial meniscus

**Fig. 4:**
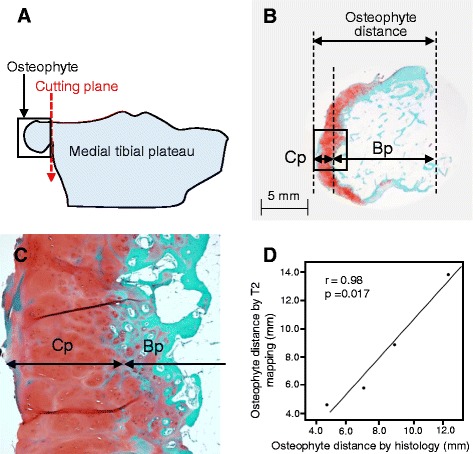
Histological analysis of medial tibial osteophyte and comparison of the osteophyte distances measured by T2 mapping and histology. **a** Schematic diagram of the surgically removed medial tibial plateau including the osteophyte. **b** Histological section of the osteophyte stained with Safranin O. **c** High-power view of the boxed area in (**b**). **d** Correlation between the osteophyte distance measured by T2 mapping magnetic resonance imaging and that by histology in patients with end-stage knee osteoarthritis. *Bp* Bone part of osteophyte, *Cp* Cartilage part of osteophyte

To examine the accuracy of T2 mapping MRI for the evaluation of osteophytes, we compared the findings of osteophytes by radiography, conventional MRI, T2 mapping MRI, and histology in patients with end-stage OA who underwent joint arthroplasty. The cartilage part of the osteophyte was undetectable by radiography (Fig. 3a and b) or difficult to be clearly shown by conventional MRI (Fig. 3c and d), but both cartilage and bone parts were detected by T2 mapping MRI (Fig. 3e and f). The osteophyte was isolated from the tibial plateau obtained at arthroplasty (Fig. 4a) and histologically examined. As shown in Fig. 4b and c, histology demonstrated that the bone part of the osteophyte is covered with cartilage cap. When the medial tibial osteophyte distance, which is the sum of the cartilage and bone parts, was separately measured by T2 mapping MRI and histology, the osteophyte distance measured by T2 mapping MRI and histology showed a direct correlation (*r* = 0.98, *p* = 0.017) (Fig. 4d).

## Discussion

In the present study, to the best of our knowledge, we have demonstrated for the first time that MME is most closely associated with the medial tibial osteophyte distance in patients with early-stage knee OA. Our data suggest a possible relationship between medial tibial osteophyte formation and MME development.

Osteophyte formation is generally thought to occur in florid stages of OA as a tissue repair process secondary to the damaged articular cartilage [7–9, 21] by following the processes of endochondral ossification [7, 9]. However, recent studies using MRI have suggested that osteophytes are commonly present within the knee joints in adults without radiographic OA [11, 16]. In this study, we have demonstrated that images of the cartilage and bone parts of osteophytes obtained by T2 mapping MRI correspond well to the histologically identified cartilage and bone parts. We have also shown that T2 mapping MRI is useful for evaluating osteophytes more accurately than conventional MRI by detecting both cartilage and bone parts. Consequently, osteophytes were found to be more frequent and larger than previously thought in patients with early-stage knee OA. Osteophytes in the medial tibial and/or femoral sites were present in 98% of patients with early-stage knee OA (49 of 50 cases). Importantly, approximately half of the whole osteophyte distance was the cartilage part, which was not detected by radiography and underestimated by conventional MRI. Our data provide direct evidence that T2 mapping MRI is applicable for measurement of whole osteophyte distance and suggest that osteophyte formation is a very common event in early-stage knee OA.

Molecular mechanisms of osteophyte formation remain unknown. However, experimental studies using animal models have shown that osteophytes develop as a result of repeated mechanical stresses [22] or by an intraarticular injection of transforming growth factor-β [7, 9, 23], bone morphogenetic protein [23], fibroblast growth factor [24], or insulin-like growth factor-1 [25]. Thus, mechanical stresses and/or soluble growth factors may contribute to osteophyte formation in early-stage knee OA joints as a different reaction from cartilage repair.

**Fig. 5:**
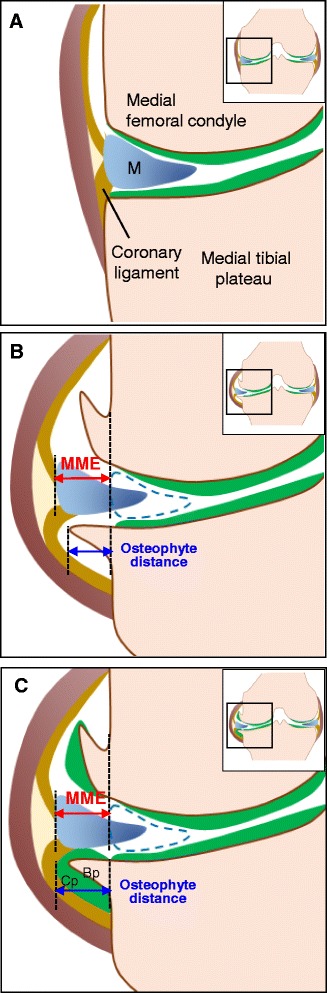
Schematic illustrations of normal knee joint and osteoarthritis knee joint with osteophyte and medial meniscal extrusion (MME). **a** Normal knee joint. Medial meniscus is tightly fixed to the coronary ligament in the medial tibial plateau. **b** Relationship between MME and medial tibial osteophyte distance based on observations by conventional magnetic resonance imaging (MRI). Note that MME distance appears to be longer than osteophyte distance. **c** Relationship between MME and medial tibial osteophyte distance according to the findings obtained by T2 mapping MRI. Note that the edge of the extruded medial meniscus matches that of the cartilage part of the osteophyte. *Bp* Bone part of osteophyte, *Cp* Cartilage part of osteophyte, *M* Medial meniscus

One of the most intriguing findings in the present study is that the medial tibial osteophyte distance was directly correlated with MME in patients with early-stage knee OA. On one hand, under normal conditions, the medial meniscus is attached tightly to the medial tibial plateau by the coronary ligament (Fig. 5a) [26]. On the other hand, the development of MRI-based studies of patients with knee OA generated the concept of MME, which denotes the medial shift of the medial meniscus [4, 6, 27, 28]. On the basis of findings obtained by conventional MRI, the edge of the medial meniscus has been considered to be more medially located than that of the medial tibial osteophyte [3], showing that the length of MME is longer than the osteophyte distance (Fig. 5b). However, as depicted in Fig. 5c, our study using T2 mapping analysis indicates that the length of MME is almost similar to the osteophyte distance, which was calculated as the sum of bone and cartilage parts detected by T2 mapping.

MME has recently attracted attention as one of the abnormalities related to the progression of knee OA [6, 20, 29–32]. Although MME seems to be enhanced dependently on the severity of knee OA and the mechanical stresses due to weight bearing [5], the mechanism of MME formation in knee OA remains unclear [33]. MME was reported to be associated with root tear of the medial meniscus [27, 34]. However, recent studies have indicated that only a few patients with knee OA with MME (2.4%) have an accompanying root tear in early-stage knee OA [35], and they have suggested that the root tear itself is caused by meniscus degeneration [36]. In the present study, the root tear was not observed in patients with early-stage knee OA, and the patients with MME grade 2 or 3, but not MME grade 0 or 1, showed direct correlation with higher T2 values of the meniscus (meniscus degradation). These data suggest that MME may occur earlier than meniscus degradation.

Although MME and medial tibial osteophyte were closely associated in the present study, their causal relationship remains obscure. One can think that osteophyte develops as a result of mechanical stresses locally induced after the occurrence of MME. In fact, researchers in a previous study proposed this hypothesis, although no direct evidence has been provided [37]. However, the medial meniscus is attached tightly to the medial plateau by the coronary ligament [26]; thus, once an osteophyte is formed, it is possible to speculate that the osteophyte may contribute to medial displacement of the meniscus, leading to MME. However, further work, such as follow-up studies of patients with early-stage knee OA by T2 mapping MRI and experimental studies with animal OA models, is definitely needed to clarify this issue.

The present study has potential limitations. We confirmed the accuracy of osteophyte evaluation by T2 mapping MRI in the combined histological analysis of the osteophytes, which were removed from end-stage knee OA joints, but such information about the osteophytes in early-stage knee OA joints is not available. In animal OA models, the osteophyte is formed through several processes, which include proliferation of synovial and resident cells in the periosteum, their differentiation into chondrocytes and formation of cartilage matrix components, further differentiation of central chondrocytes into hypertrophic chondrocytes, bone formation through endochondral ossification, and fully developed osteophyte formation [7]. A similar mechanism has been proposed for human osteophyte formation [38], although to the best of our knowledge, there are no reports describing the processes of human osteophyte formation. Therefore, further analyses of the knee joints of human subjects, including cadavers, are necessary to clarify the mechanism of osteophyte formation and the relationship between the T2 mapping MRI data and pathological changes of osteophytes. Ultrasonography has been introduced to examine OA changes of knee joints and reported to be more sensitive than radiography to detect knee osteophytes [39] and MME [40]. Cartilage is exhibited as a hypoechoic area on ultrasonograms [41], and the cartilage thickness is underestimated by ultrasonography compared with conventional MRI [42, 43]. However, because ultrasonography is easy to use and available at a much lower cost than MRI, frequent monitoring of MME and osteophytes by ultrasonography is possible, and this may provide a clue leading to better understanding of the relationship between MME and osteophytes. Although this study provides the first evidence of the close relationship between medial tibial osteophyte distance and MME, this was based on MRI data of subjects in a supine, non-weight-bearing position, which may lead to an underestimation of what would be expected in axially loaded knees. Thus, to further analyze the relationship between MME and osteophyte formation, the degree of MME should be assessed with the knee bearing a full load, the images of which can be obtained by the recently developed upright positional MRI.

## Conclusions

We have demonstrated that the medial tibial osteophyte is commonly observed in patients with early-stage knee OA, showing a close association with MME, and that MME is positively correlated with medial meniscal degeneration.

## Abbreviations

BMI: Body mass index
Bp: Bone part of osteophyte
Cp: Cartilage part of osteophyte
FOV: Field of view
ICC: Interclass correlation
K/L: Kellgren-Lawrence classification
M: Meniscus
MFTJ: Medial tibiofemoral joint
MME: Medial meniscal extrusion
MRI: Magnetic resonance imaging
OA: Osteoarthritis
TE: Echo time
TR: Repetition time
WORMS: Whole-Organ Magnetic Resonance Imaging Score
